# Early stent thrombosis confirmed in a cancer patient receiving regorafenib, despite triple antithrombotic therapy: a case report

**DOI:** 10.1186/s12872-021-01888-9

**Published:** 2021-01-30

**Authors:** Keisuke Shoji, Kan Zen, Takashi Ookura, Kenji Yanishi, Satoaki Matoba

**Affiliations:** Department of Cardiovascular Medicine, Kyoto Prefectural University of Medicine, 465 Kajii-cho Kawaramachi-Hirokoji, Kamigyo-ku, Kyoto, 602-8566 Japan

**Keywords:** Stent thrombosis, Cancer, Coronary artery disease, Regorafenib, Vascular endothelial growth factor

## Abstract

**Background:**

While developments in oncology have lengthened survival in patients with cancer, such patients often develop cardiovascular diseases. Thus, percutaneous coronary intervention (PCI) is frequently undertaken in them. Although stent thrombosis remains a fatal complication in stent-based PCI, worldwide consensus panels tend to recommend shorter duration of dual-antiplatelet therapy. This is based on its clinical efficacy that has resulted from technological innovation. However, there is insufficient discussion on the risk of stent thrombosis in cancer patients with coronary artery disease, especially in those undergoing chemotherapeutic regimens that have a risk for thrombosis, such as regimens with the anti-vascular endothelial growth factor. Presented here is a case of early stent thrombosis that occurred in a cancer patient on regorafenib, despite the administration of triple antithrombotic therapy.

Case presentation

A 66-year-old Japanese male patient received regorafenib for metastatic colorectal carcinoma and apixaban for deep vein thrombosis. Coronary angiography revealed severe stenosis in the proximal left anterior descending artery. A sirolimus-eluting stent was implanted, without malapposition and under-expansion, under intravascular ultrasound guidance while administering a triple antithrombotic therapy (aspirin: 100 mg/day, prasugrel: 3.75 mg/day, and apixaban: 5 mg/day). However, he was admitted to the hospital for exacerbation of heart failure 1 month after PCI. Coronary angiography revealed contrastive defects in the previous stent. Optical frequency domain imaging confirmed stent thrombosis. PCI was successfully performed with perfusion balloon long-inflation. Antithrombotic therapy was enhanced (aspirin: 100 mg/day, ticagrelor: 120 mg/day, and apixaban: 10 mg/day) and regorafenib was discontinued permanently. While ischemic events did not occur thereafter, the patient died due to metastatic carcinoma progression.

**Conclusions:**

This case suggests that anti-vascular endothelial growth factor might contribute to early stent thrombosis, despite triple antithrombotic therapy. Further discussion is needed on the surveillance and management of cancer patients with coronary artery disease receiving chemotherapy, which carries a risk of thrombosis.

## Background

Cardio-oncology is a rapidly growing subspeciality worldwide. Recent oncological developments have led to favorable clinical outcomes in cancer patients; however, many cancer survivors develop cardiovascular diseases. As a result, percutaneous coronary intervention (PCI) is frequently performed in cancer patients, who also develop coronary artery disease (CAD) while receiving chemotherapy.

Stent thrombosis (ST) is a rare but serious complication of PCI. Malignant disease is known as a strong risk factor for the development of ST and hemorrhage after stent implantation [1]. In addition, many modern chemotherapies are associated with vascular complications such as thrombosis and major bleeding [2]. Cancer and its treatment with chemotherapy are associated with a potential risk of thrombotic and bleeding complications in patients undergoing PCI. However, currently, there is insufficient evidence for optimal prevention and risk stratification of ST in these patients.

Described below is a case of early ST that was confirmed by optical frequency domain imaging (OFDI). In this case, ST occurred in a cancer patient receiving anti-vascular endothelial growth factor (VEGF) therapy in our hospital despite the administration of a triple antithrombotic therapy.

## Case presentation

A 66-year-old Japanese male patient had received regorafenib (160 mg/day) as an anticancer treatment for metastatic colorectal carcinoma. His medical history included hypertension, diabetes mellitus, dyslipidemia, chronic heart failure, deep vein thrombosis (DVT), and permanent pacemaker implantation due to complete atrioventricular block. His DVT had been managed with low-dose apixaban (5 mg/day; body weight: 75 kg, creatinine: 1.0 mg/dL) to avoid bleeding complications.

**Fig. 1:**
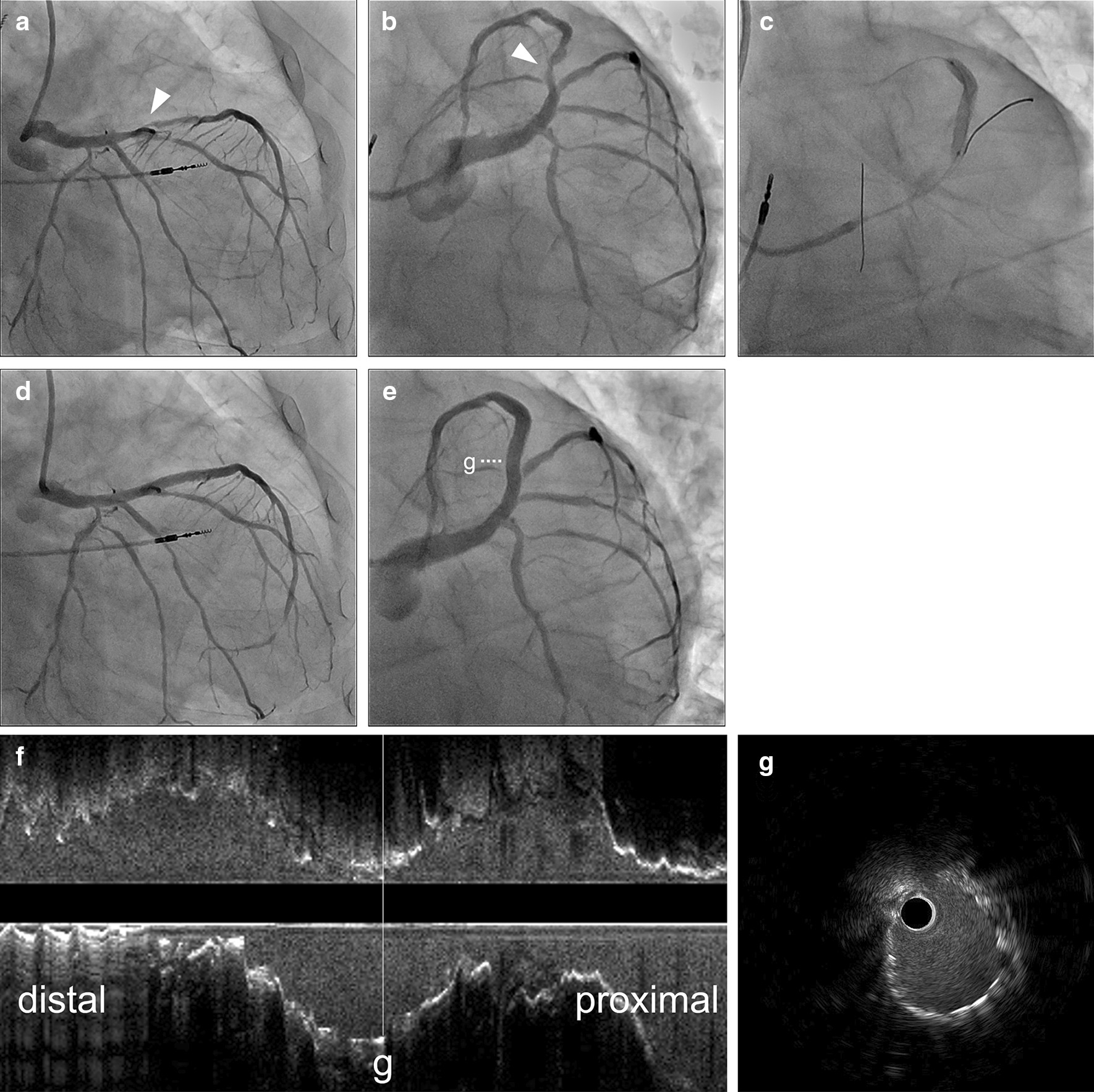
Percutaneous coronary intervention for left anterior descending artery under intravascular ultrasound guidance. **a**, **b** Severe stenosis in the left anterior descending (LAD) artery (arrowhead) on initial coronary angiography (CAG). **c** Stent implantation in the LAD. **d**–**g** Final CAG showing an optimal result, with longitudinal and cross-sectional views in intravascular ultrasound imaging; acceptable stent expansion is confirmed

**Fig. 2:**
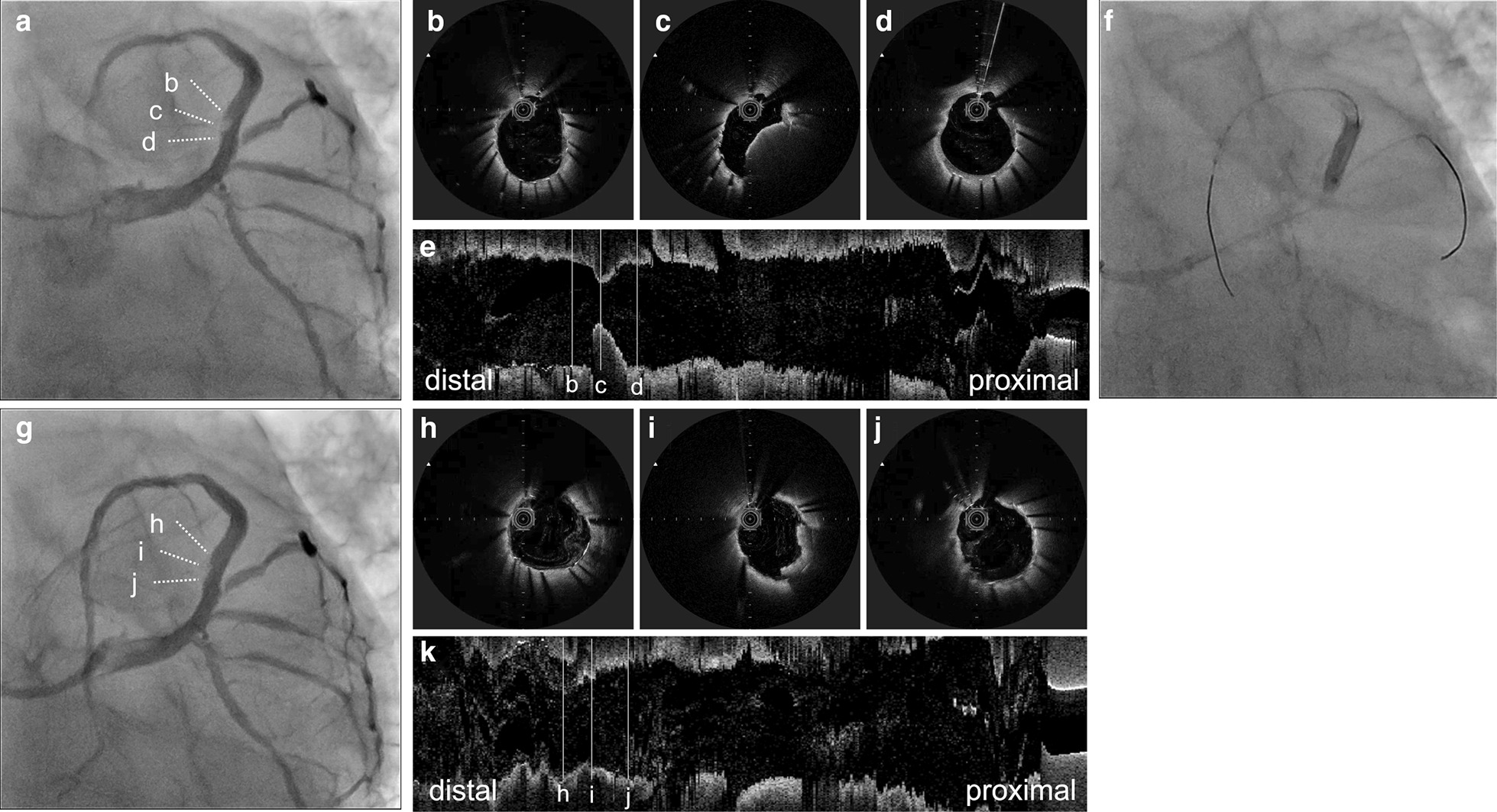
Early stent thrombosis and revascularization. **a** Focal and eccentric contrastive defect in the previous left anterior descending stent. **b**–**e** Cross-sectional and longitudinal views of the stent thrombosis in optical frequency domain imaging (OFDI). **f** 3.5-mm perfusion balloon is dilated for 3 min. **g** Final coronary angiography reveals reduction of the contrastive defect and thrombolysis in myocardial infarction flow grade 3 without distal emboli. **h**–**k** Cross-sectional and longitudinal views in OFDI showing an increase in the lumen area and a small amount of residual thrombus

Two months ago, he had suffered from non-ST-elevation myocardial infarction with the high-lateral (HL) branch as the culprit lesion, accompanied by chest discomfort; the HL branch occlusion was treated with balloon dilation only to avoid the need of a long-term triple antithrombotic therapy. At the same time, coronary angiography (CAG) revealed severe stenosis in the proximal left anterior descending (LAD) artery. A fractional flow reserve value of 0.72 confirmed a physiologically significant lesion. After PCI for the HL branch, regorafenib was withdrawn temporarily, and a triple antithrombotic therapy (aspirin: 100 mg/day, prasugrel: 3.75 mg/day, and apixaban: 5 mg/day) was successfully continued for two months without bleeding complications. Subsequently, the patient underwent PCI for severe stenosis in the LAD lesion two months after PCI for HL, as he demonstrated good tolerance to the triple antithrombotic therapy. Other medications administered included bisoprolol (1.25 mg/day), enalapril (2.5 mg/day), furosemide (20 mg/day), esomeprazole (20 mg/day), amlodipine (5 mg/day), sitagliptin (50 mg/day), mitiglinide (30 mg/day), and insulin degludec (9 units). In addition, routine blood testing revealed an elevated level of B-type natriuretic peptide (BNP), normal kidney function, high value of HbA1c, and mild anemia (BNP: 191.6 pg/mL, creatinine: 0.68 mg/dL, estimated glomerular filtration rate: 89.3 mL/minute/1.73 m^2^, HbA1c: 8.4%, hemoglobin: 9.9 g/dL, and platelet count: 318 × 10^3^/µL). He was classified as having a high thrombotic and bleeding risk at this time, according to the CREDO-Kyoto thrombotic and bleeding risk scores. A sirolimus-eluting stent, which is a drug-eluting stent (DES) (Ultimaster Tansei 3.5/38 mm; Terumo Corporation, Tokyo, Japan), was implanted in the LAD lesion without malapposition or significant under-expansion, as confirmed by intravascular ultrasound imaging (IVUS) analysis (Fig. 1). After PCI for the LAD, regorafenib was resumed because it exhibited a clinical effect on the patient's cancer. Nevertheless, the patient was admitted to our hospital for congestive heart failure with dyspnea one month later (BNP: 1,512.5 pg/mL). Echocardiography demonstrated new severe hypokinesis of the anteroseptal wall and apex. Despite continued triple antithrombotic therapy, CAG revealed focal and eccentric contrastive defects in the previous LAD stent, and OFDI confirmed ST (Fig. 2a–e). A 3.5/20 mm perfusion balloon (Ryusei, Kaneka Medical, Tokyo, Japan) was inflated for 3 min using an embolic protection device (Fig. 2f). CAG and OFDI confirmed optimal initial gain in the ST site and thrombolysis in myocardial infarction flow grade 3, with no complications including distal emboli (Fig. 2g–k). Antithrombotic therapy was further enhanced temporarily to prevent the recurrence of ST; it included aspirin (100 mg/day), ticagrelor (120 mg/day), and apixaban (10 mg/day). The administration of regorafenib was discontinued after PCI for ST in the LAD, and aspirin and ticagrelor were discontinued 1 and 2 months later, respectively. No further thromboembolic events (including definite/probable ST) were observed, and heart failure was managed well. However, he died due to the progression of metastatic colorectal carcinoma about 3 months after ST onset.

## Discussion and conclusions

In this modern stent-based PCI era, ST represents a fatal and life-threatening complication. According to previous reports, the risk of ST is strongly associated with both, the patient background and procedural aspects [1, 3]. Certain patient background factors are independent predictors of a bleeding risk; malignant disease is a strong risk factor for major bleeding after PCI, as well as ST [1]. Our patient was classified as having a high thrombotic (4 points: anemia, heart failure, diabetes mellitus) and bleeding (4 points: heart failure, prior myocardial infarction, malignancy) risk, according to the CREDO-Kyoto thrombotic and bleeding risk scores [3]. Thus, we always had to consider the tradeoffs between the thrombotic and bleeding risks and PCI while caring for him.

The most frequent optical coherence tomography findings in early ST (within 30 days after index PCI) are uncovered struts and under-expansion [4]. Incomplete neointimal coverage of the struts in the early phase is normal, but the risk of ST associated with this procedure needs to be minimized. This risk could be reduced by optimizing stent placement with intracoronary imaging techniques and developing newer DES technology [1]. Moreover, dual-antiplatelet therapy (DAPT) with aspirin and a P2Y_12_ receptor blocker after coronary stent implantation has been effective in preventing ST, and has been the standard treatment. Regarding P2Y_12_ receptor blockers, prasugrel or ticagrelor, in combination with aspirin, has been associated with a greater reduction in ST as compared to a combination of clopidogrel and aspirin [5, 6]. Furthermore, among stented patients with acute coronary syndrome treated by DAPT, low-dose rivaroxaban administration has been associated with a reduction in ST [7]. Thus, antithrombotic therapy after PCI has been established worldwide, but the optimal duration of DAPT remains controversial. Recent global guidelines recommend a shorter duration of DAPT for clinical efficacy. Additionally, it is recommended that the duration of the combination of triple antithrombotic therapy with DAPT and oral anticoagulants be as brief as possible [8]. However, there remains a lack of discussion regarding an appropriate antiplatelet therapy after PCI for cancer patients who are or are not undergoing chemotherapy. In our patient, although new-generation DES implantation was optimized by IVUS and a more potent antithrombotic therapy (DAPT and direct oral anticoagulant [DOAC]) was continued, early ST still occurred. This suggests that a standardized antithrombotic drug reduction may cause life-threatening ST in cancer patients receiving chemotherapy. The patient in our case was managed by changing the P2Y_12_ receptor blocker, increasing the DOAC dose, and withdrawing regorafenib until an adequate neointimal coverage of the struts was achieved. Thus, the risk of ST and the optimal duration of DAPT in cancer patients undergoing chemotherapy should be explored further.

While the association between venous thromboembolism (VTE) and malignancy is well-discussed, arterial thrombosis has more recently been recognized as a serious complication of cancer and chemotherapy. One study, based on a large database in the USA, reported that the incidence of arterial thromboembolic events (ATEs) in patients with cancer at 6 months was 4.7% [9]; patients with lung, gastric, or pancreatic cancers had the highest rates of ATEs (8.3, 6.5, and 5.9%, respectively). Moreover, an advanced stage of cancer was associated with a significant increase in the incidence of ATEs (stage 0 vs. stage 4: 2.3% vs. 7.7% at 6 months). Furthermore, certain chemotherapeutic agents, including VEGF inhibitors, have been reported to be associated with ATEs [10]; cisplatin, nilotinib, ponatinib, 5-FU, and capecitabine are particularly associated with a high incidence of coronary artery thrombotic events. Thus, while determining the risk of ischemic events after PCI, physicians should take into account the type of cancer, progression, and chemotherapeutic regimen. Similarly, the risks of occurrence and recurrence of cancer-associated VTE were found to vary according to the type and spread of the malignant disease [11]. Recent clinical trials assessing DOACs for cancer-associated VTE reported that DOACs seemed to be a reasonable treatment for VTE in such patients [12]. However, it should be noted that in these trials, bleeding events occurred in cases with gastrointestinal and urological tumors; such an occurrence of bleeding events in different types of cancers may also apply to DAPT after PCI in cancer patients. The traditional stratification of risks for bleeding and ischemic events after coronary stent implantation may need to be supplemented with information on the cancer type, progression, and chemotherapeutic regimen.

Regorafenib, an oral multi-targeted receptor tyrosine kinase inhibitor that targets VEGF (thereby affecting cell proliferation and angiogenesis), has been indicated for metastatic colorectal carcinoma. However, it is associated with cardiovascular complications such as hypertension, hemorrhage, thrombosis, and heart failure [2, 10, 13]. Regorafenib was previously reported to be less involved in thrombosis as compared to similar drugs, and has been relatively well-tolerated by patients [13]. However, increased platelet activation, endothelial dysfunction, and dysfunctional nitric oxide metabolism due to VEGF inhibitors result in thrombosis, because VEGF is an important signaling factor for endothelial cell health, which is essential for blood vessel formation and maintenance [10]. Thus, VEGF is important for endothelial cell health and vascular repair in patients undergoing PCI. As a result, VEGF inhibitors, such as regorafenib, might be associated with ST. In our case, it was important to consider a change in the chemotherapeutic regimen before PCI. However, the patient wished to continue regorafenib, because it was an almost final-line treatment for his cancer. Oncology and cardiovascular experts should make every effort to provide optimal medical care while respecting the patients' wishes.

The management of hemodynamically significant CAD in cancer patients, especially those undergoing chemotherapy, has not been fully established. Optimal stent replacement during PCI under intracoronary imaging could reduce the risk of ST, but this may be insufficient. The optimal treatment strategy for cancer patients with CAD, including invasive revascularization, antithrombotic therapy, and chemotherapy, needs to be verified. Future developments in the field of cardio-oncology will need to adapt to changes in the patient population and focus on optimal prevention and surveillance strategies accordingly.

## Data Availability

All data generated or analyzed during this study are included in this published article and in its additional files.

## Abbreviations

ATEs: arterial thromboembolic events
BNP: B-type natriuretic peptide
CAD: coronary artery disease
CAG: coronary angiography
DAPT: dual-antiplatelet therapy
DES: drug-eluting stent
DOAC: direct oral anticoagulant
DVT: deep venous thrombosis
HL: high-lateral
OFDI: optical frequency domain imaging
IVUS: intravascular ultrasound imaging
LAD: left anterior descending
PCI: percutaneous coronary intervention
ST: stent thrombosis
VEGF: vascular endothelial growth factor
VTE: venous thromboembolism
